# Primary Hyperparathyroidism in Pregnancy—A Rare Cause of Life-Threatening Hypercalcemia: Case Report and Literature Review

**DOI:** 10.1155/2011/520516

**Published:** 2011-07-18

**Authors:** S. Malekar-Raikar, B. P. Sinnott

**Affiliations:** ^1^Department of Medicine, Mercy Hospital and Medical Center, Chicago, IL 60616, USA; ^2^Section of Mineral Metabolism, UTSW at Dallas, Dallas, TX 75390-8885, USA

## Abstract

*Objective*. To report a case of primary hyperparathyroidism in a pregnant patient, report the obstetric and neonatal outcomes, and review the relevant literature. *Results*. A 29-year-old primigravida was successfully treated for PHP with minimally invasive resection of a parathyroid adenoma in the second trimester of pregnancy. A healthy baby girl was delivered at 37-week gestation with an unremarkable neonatal course. To the best of our knowledge, this is the second case report in the literature utilizing intraoperative PTH during a parathyroidectomy in a pregnant woman. *Conclusions*. Primary hyperparathyroidism is a rare life-threatening condition that can present during pregnancy. The diagnosis can be difficult to establish during pregnancy, given the nonspecific symptoms related to hypercalcemia. However, a better understanding of the condition, improved diagnostic studies, and well-organized multidisciplinary management decisions can significantly reduce the morbidity and mortality associated with the disease during pregnancy. This case report is presented to highlight the value of early diagnosis and appropriate management of PHP during pregnancy.

## 1. Introduction

Primary hyperparathyroidism (PHP) is the most common cause of hypercalcemia seen in the outpatient setting with a prevalence of 0.15% in the general population [1, 2]. Women are affected with PHP 3 times more commonly than men [1]. The occurrence of PHP during pregnancy is a rare event, with less than 200 cases reported in the English literature. The incidence of PHP in pregnant reproductive age women is reported to be 8/100,000 population/year [1]. The most common cause of primary hyperparathyroidism in pregnancy is a single adenoma, representing 85% of all cases, followed by 10% from primary parathyroid hyperplasia, 3% from multiple adenomas, and 2% from parathyroid cancer [2, 3]. Between 23 and 80% of patients with PHP are asymptomatic [1, 4]. Symptoms associated with hypercalcemia are often variable and vague in nature leading to a potential delay in the diagnosis and management of this important disorder during pregnancy [5, 6]. The pregnant state presents a challenge to the diagnosis of hypercalcemia related to the many physiological changes that occur [2, 7, 8]. In fact, the most common indicator of hyperparathyroidism during a pregnancy is the development of neonatal tetany [9–13]. Maternal complication rates related to PHP during pregnancy have been reported to be as high as 67% [13], and fetal complications are reported to occur in up to 80% cases [14]. Early recognition of the condition, followed by appropriate management and treatment, has been shown to significantly reduce these complications. Several different medical regimens have been tried with varying degrees of success. As exemplified by our case, it is recommended that women with PHP during pregnancy should undergo parathyroidectomy during the second trimester to minimise complications [15, 16].

## 2. Case Report

A 29-year-old primigravida (G1P0A0) African American woman presented during her 18th week of gestation with a one-day history of nausea and vomiting. She had experienced an unremarkable pregnancy up until this time. She denied any history of abdominal pain, dysuria, constipation, polydipsia, polyuria, weight loss, anorexia, or muscle weakness during her pregnancy. She had no history of calcium disorders, kidney stones, fractures, osteoporosis or endocrinopathies. Family history was negative for calcium disorders, kidney stones, fractures, osteoporosis, endocrinopathies or parathyroid disorders. She was taking prenatal multivitamins as prescribed. She was not taking thiazide diuretics, antacids, or lithium which could influence her calcium status. 

On physical examination, she was well nourished and in no distress. Her blood pressure was 110/68 mmHg, heart rate 60/min and regular, respiratory rate 18/minute, and weighed 295 pounds (134 kg) with a height of 62 inches (157.5 cm). Examination of her neck revealed a 30 gm goiter without any nodules or bruits. There were no palpable neck masses, jaw abnormalities, or cervical or supraclavicular lymphadenopathy. On abdominal examination, there was evidence of a palpable uterus up to 2 cm below the umbilicus, consistent with an 18-week gestation. There was no edema of her extremities. She had no kyphosis or bone tenderness. The remainder of the systemic examination was unremarkable. 

Initial laboratory evaluation revealed severe hypercalcemia with a calcium level 13.9 mg/dL (reference range 8.5–10.3), albumin 3.3 g/dL (3.2–5.5), and a phosphorous level 2 mg/dL (2.4–4.1). She had normal renal function with a low potassium level 3.0 mmol/L (3.5–5.0), related to emesis. Her 1,25-dihydroxycholecalciferol level was 94.3 ng/mL (reference range 15.9–55.6), 25-hydroxyvitamin D level 40 ng/mL (8.9–46.7), and parathyroid-hormone-related peptide (PTHrp) was <0.3 pmol/L (0.0–1.5). EKG showed right bundle branch block with a normal QT interval. An endocrine consult was ordered in view of her significant hypercalcemia. On further workup, she was found to have an elevated PTH level 261.8 pg/mL (16–48 pg/mL) and a urinary calcium level 479 mg/24 hr (reference range 100–300 mg/24 h) confirming a diagnosis of primary hyperparathyroidism and ruling out familial hypocalciuric hypercalcemia. Laboratory workup was inconsistent with vitamin D intoxication, milk alkali syndrome, or malignancy. She was biochemically euthyroid.

**Figure 1:**
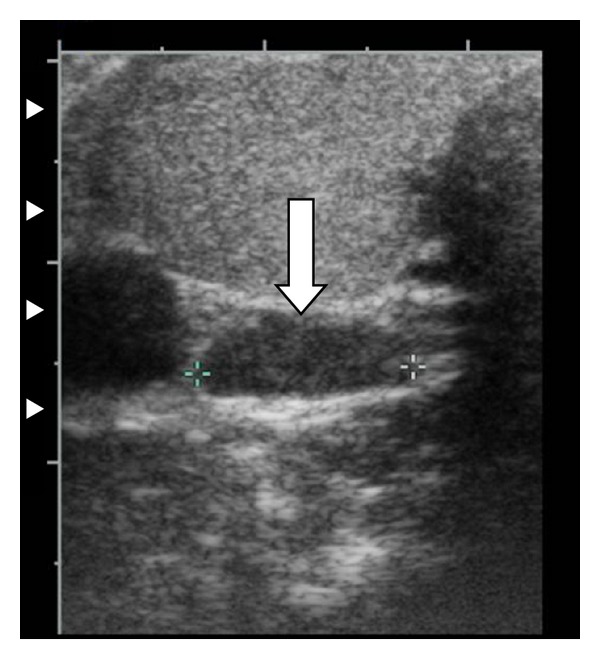
Ultrasound image demonstrates a hypoechoic mass, 1.9 × 0.4 × 1 cm, inferior and posterior to the right lobe of the thyroid, consistent an enlarged right inferior parathyroid gland.

Ultrasonography of her neck revealed a hypoechoic nodule suggestive for an enlarged right inferior parathyroid gland measuring 1.9 × 0.4 × 1 cm. It was seen just deep to the inferior aspect of the right lobe of the thyroid gland (Figure 1). An obstetrical ultrasound confirmed a live single intrauterine gestation consistent with 18-week of gestation.

**Figure 2:**
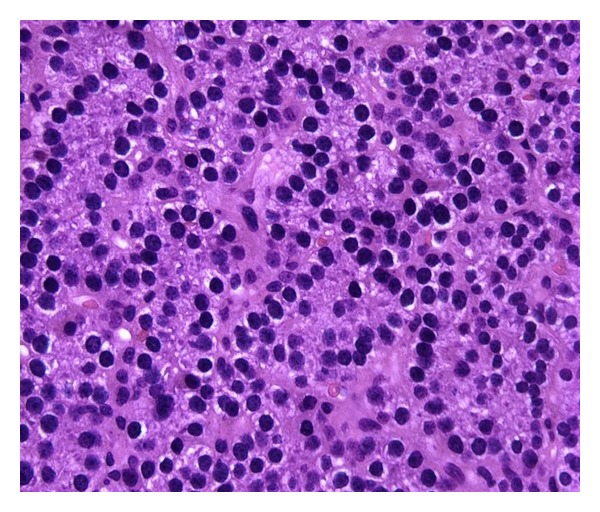
H&E staining; magnification ×40; demonstrates a parathyroid adenoma composed of chief cells with some oxyphil cells in a delicate capillary network.

She was initially managed with conservative measures namely, a eucalcemic diet and aggressive hydration with minimal improvement in her clinical or biochemical status. Due to her persistent symptomatic hypercalcemia despite conservative measures, the decision was made to perform an elective parathyroidectomy of the enlarged parathyroid gland. She underwent a minimally invasive right inferior parathyroidectomy at 18-week gestation. There was a marked drop in the intraoperative PTH levels from 203 pg/mL initially to 28.5 pg/mL at 10 minutes after the removal of the enlarged parathyroid gland, confirming a successful operation. Postoperative calcium and PTH levels were 9.4 mg/dL and 28.5 pg/mL, respectively. Pathological evaluation of the resected mass demonstrated a parathyroid gland weighing 700 mgs. Histopathologic examination of the parathyroid gland was consistent with a parathyroid adenoma (Figure 2). The patient was discharged home on postoperative day number one with a normal calcium level. The remainder of her pregnancy was uncomplicated until week 37 of her gestation. 

At 37-week gestation, induction of labor was necessary due to preeclampsia. Her corrected calcium level at that time was 10.3 mg/dL. The patient delivered a healthy baby girl weighing 5 pounds and 11 ounces (2.27 kg) with APGAR scores at both 1 and 5 minutes of 9/10. The baby was normocalcemic with a calcium level of 8.8 mg/dL and a PTH level of 109 pg/mL on day 2. The mother and baby were discharged home on the third day after delivery. 

The patient remained normocalcemic 1 year post-op with a parathyroid level of 54 pg/mL.

## 3. Discussion

PHP as a cause of hypercalcemia is typically a benign disease of the elderly. However, when present during pregnancy, it may be a rare cause of life-threatening hypercalcemia. Maternal complication rates related to PHP during pregnancy have been reported to be as high as 67% [13], and fetal complications are reported to occur in up to 80% cases [14]. Early identification and management of this condition in pregnancy can significantly reduce fetal, neonatal, and maternal morbidity and mortality associated with this important condition.

### 3.1. Calcium Homeostasis during Pregnancy

During pregnancy, maternal calcium homeostasis adapts to provide calcium for the developing fetus. Many of the physiological changes associated with pregnancy present a challenge to the diagnosis of hypercalcemia, namely, hemodilution related to intravascular fluid expansion, an increase in glomerular filtration rate resulting in maternal hypercalciuria [17] and gestational hypoalbuminemia [18]. A physiologic fall in serum albumin leads to a fall in total calcium levels; however, ionized calcium levels are similar to the nonpregnant state [19]. The lower levels of total serum calcium seen in pregnancy may mask mild hypercalcemia if correction for a lower albumin is not performed [20]. 

The increased calcium demands during pregnancy are primarily meet via the enhanced maternal intestinal calcium absorption [21] related to increased Vit D1,25OH production [17], with a 2-fold increase in intestinal calcium absorption occurring during pregnancy [22]. In our patient, 1,25-dihydroxycholecalciferol levels were nearly twice the upper limit of the healthy subject range. Biologically active 1,25-dihydroxycholecalciferol is elevated 2-fold as a result of the increased activity of maternal 1 *α* hydroxylase activity under the regulation of PTHrp, prolactin, estradiol, and human placental lactogen rather than direct stimulatory effects of intact PTH [2, 23–25].

A term infant requires about 25–30 gms of calcium for mineralization of its bones during fetal development [23]. Approximately 80% of this calcium accumulation occurs during the third trimester of pregnancy. Calcium transfer occurs across a placental-fetal calcium gradient of 1.0 : 1.4 throughout pregnancy [2]. The fetal blood has a higher concentration of calcium compared to maternal blood resulting in suppression of fetal parathyroid development until after delivery [26]. At birth, the neonate has relative hypercalcemia and suppressed PTH levels. 

In maternal primary hyperparathyroidism, the calcium gradient is further elevated, resulting in a state of profound fetal parathyroid gland suppression leading to a hypocalcemic state because of an inability to mobilize calcium from bone and even fetal tetany in the neonatal period [12, 15]. Neonatal calcium levels reach a nadir at 6–12 hours postdelivery, related to parathyroid gland suppression and the sudden loss of placental calcium transport, followed by a gradual increase in the calcium level towards the adult range over the following 7–14 days, as the parathyroid glands recover [2, 23]. Fetal PTHrp and PTH participate in the regulation of fetal calcium homeostasis [23, 27].

### 3.2. Diagnostic Workup

Routine prenatal testing does not screen for PHP. A diagnosis of PHP in pregnancy should be considered with the simultaneous findings of an elevated total corrected calcium level (>9.5 mg/dL) or ionized calcium level, hypophosphatemia (<2.5 mg/dL), and an elevated serum PTH level in the absence of other causes of hypercalcemia [18]. Additionally, the diagnosis should be considered in any patient who presents with the classic features of hyperparathyroidism such as pancreatitis, fractures, or peptic ulcer disease (PUD) [28]. It is necessary to exclude familial hypocalciuric hypercalcemia, a benign inherited calcium disorder, by quantifying an inappropriately low/normal 24 urinary calcium excretion in the setting of a high serum calcium level, high/normal PTH, and a normal serum vitamin D25OH level. During the evaluation of PHP in a young woman, the possibility of hereditary syndromes should be considered, namely, MEN-1, MEN-2, familial parathyroid hyperplasia syndromes, or jaw-tumor syndrome.

Ultrasonography of the neck is the investigation of choice during pregnancy for localization of parathyroid adenomas with a sensitivity of 69% and a specificity 94% [29]. The majority of parathyroid adenomas occur in the inferior parathyroid glands, as in our case. Computerized tomography and sestamibi scintigraphy for the detection of parathyroid adenomas are contraindicated during pregnancy due to the possible risks of ionizing radiation to the fetus. MRI of the neck can be safely used during pregnancy. 

### 3.3. Maternal Complications Associated with Primary Hyperparathyroidism

PHP has been reported to lead to maternal complications in 2/3 of cases [13]. The presentation of hypercalcemia in pregnancy related to PHP is variable and ranges from asymptomatic in 23% to symptoms such as nausea, vomiting, and anorexia in 36%, weakness and fatigue in 34%, and neurological/psychiatric manifestations in 26% based on a review of 70 women with PHP during pregnancy [4]. Other reports suggest that up to 80% of patient with hyperparathyroidism may be asymptomatic [1, 4]. 

Nephrolithiasis (24–36%) is the most common maternal complication followed by bone disease and pancreatitis (7–13%) [4, 28, 30–33]. Pancreatitis is considered an ominous sign of disease severity and can occur simultaneously with a hypercalcemic crisis [4, 28, 30–33]. Other maternal complications include hyperemesis gravidarum, preeclampsia, tremors, fractures, constipation, depression, blurred vision, uremia, seizures, and coma [6, 13, 30, 32, 34]. A literature review of PHP in pregnancy suggested that PHP should be considered a risk factor for eclampsia [30], as seen in our patient. 

Interestingly, in the largest retrospective series of PHP gestations published to date, 2/3 of study participants had a prior history of miscarriage, with a 100% successful pregnancy rate after parathyroidectomy. In the same review, pregnancy loss in patients with untreated PHP occurred typically in the late 1st or early 2nd trimester, with second trimester losses being 6-fold higher than expected and miscarriage rates increased with increasing maternal serum calcium levels [15]. 

The most feared and life-threatening complication, a hypercalcemic crisis, can be seen with serum calcium levels >14 mg/dL and is characterised by nausea, vomiting, tremors, dehydration, and mental status changes which can progress to uremia, coma, and even death [4, 11, 20, 30, 31, 33, 35]. A hypercalcemic crisis can also present in the early postpartum period due to the sudden interruption of the transplacental shunting of calcium from the mother to the fetus [6, 13, 36]. A hypercalcemic crisis has been associated with a reported 25% perinatal mortality rate and a 50% frequency of neonatal tetany [10, 37].

### 3.4. Fetal Complications Associated with Maternal Hyperparathyroidism

Fetal complications have been reported to be as high as 80% in mothers with PHP who had not received appropriate treatment [14]. The most serious fetal complications are neonatal tetany, still birth, and miscarriage [15, 28]. Shangolds analysed 63 cases of PHP during pregnancy in the early eighties and reported a 45% neonatal complication rate and a 46% perinatal complication rate, with tetany being the primary cause of morbidity. Perinatal death occurred in nearly 25% of cases. Their 30-year review did find however that perinatal morbidity and mortality had decreased over time [11]. 

Up to 50% of neonates born to mothers with untreated PHP may have neonatal hypocalcemia [10, 11] and are at risk for tetany because of suppressed parathyroid gland development [12]. Fetal complications include premature birth, intrauterine growth retardation [10], low birth weight, transient hypoparathyroidism, or even fetal demise (27–31%) [14, 16, 38]. Hypocalcemia resulting from impaired fetal parathyroid gland development in utero [2] is usually transient and resolves with treatment within 3–5 months [11]; however, there are case reports of the development of permanent hypoparathyroidism [11, 38–40].

Over time, there has been increased awareness and improved management of PHP, with a reduction in still births and/or neonatal deaths from maternal PHP from 27% (1963–1975) to 5% (1976–1990); however, the incidence of neonatal tetany has remained high at 46% [16]. Therefore, PHP during pregnancy remains a serious disorder. Fortunately, in appropriately managed cases, fetal complications can be reduced by a factor of four [11, 16].

### 3.5. Management of Hyperparathyroidism

In 2009, the third international workshop published revised evidence-based guidelines regarding the management of patients with primary hyperparathyroidism [41]. Unfortunately, given the rarity of PHP in pregnancy, these guidelines do not include any official recommendations for pregnant women; however, there are management recommendations published elsewhere in the literature [6]. Management of hyperparathyroidism during pregnancy needs to be individualized and should take into account the patient's symptoms, the severity of the hypercalcemia, the gestational age of the fetus, and the reliability of the mother for close outpatient followup. 

#### 3.5.1. Asymptomatic Patients

and patients with mild total corrected hypercalcemia (<12 mg/dL) can be reasonably managed with conservative medical management such as oral hydration, low calcium intake, and close fetal surveillance [6]. Several different medical management options have been used with varying degrees of success, namely, an eucalcemic diet, oral phosphates, and furosemide, a category C drug during pregnancy. Calcitonin, a category B drug, which lowers calcium acutely via direct inhibition of osteoclast function can be used safely in pregnancy [6, 30]. Patients often require aggressive intravenous fluid replacement as they may be dehydrated from hypercalcemic-induced nephrogenic diabetes insipidus. Intermittent parenteral furosemide to promote calciuresis may be necessary. The goal of medical management is to lower the serum calcium level to below 12 mg/dL [6, 20], without deleterious effects; however, the complication rates for medically treated patients are high. The development of a hypercalcemic crisis is one of the most serious complications and is characterised by a calcium level >14 mg/dL and the development of prerenal failure. Medically treated patients must be carefully monitored for a hyperparathyroid crisis during pregnancy and in the postpartum period as loss of placental calcium transfer may aggrevate the underlying hypercalcemia. If patients decide on continued conservative management throughout pregnancy then close perinatal and maternal surveillance is critical [42] to minimize significant risks. During this time, if the patient develops hypercalcemia or becomes symptomatic despite medical management, then surgical intervention should be strongly advised regardless of the advanced gestational age.

#### 3.5.2. Symptomatic Patients

with calcium levels greater than 12 mg/dL during any trimester require immediate hospitalization, treatment with aggressive hydration, and assessment of fetal well-being, as in our case [15]. The management of severe or refractory hypercalcemia may require acute short-term hemodialysis [43]. If all medical measures fail, urgent parathyroidectomy may be recommended regardless of the gestational age of the fetus. 

Importantly, none of the above-mentioned medical options have been proven to have an advantage over surgical management of a parathyroid adenoma. Indeed, the risk of obstetric complications is significantly greater in woman who underwent medical rather than surgical treatment [11, 16]. A review comparing 70 medically managed patients with 39 patients who had undergone parathyroidectomy during pregnancy by Kelly [16] demonstrated that medically managed patients had a neonatal complication rate of 37% and neonatal mortality of 16%, compared to 10% and 3%, respectively, in those treated surgically. Additionally, Schnatz et al. [30] showed up to a 23.5% incidence of clinically significant fetal complications from medically treated maternal PHP compared with an 11.8% incidence of clinically significant fetal complications from maternal parathyroidectomy during pregnancy. Regarding maternal complications, clinically significant complications related to PHP were as high as 25% in the medically managed patients compared to 12.5% in the surgically managed patients. These findings suggest that surgical intervention is superior to conservative management during pregnancy.

A minimally invasive parathyroidectomy during the second trimester is the therapeutic gold standard and the most definitive strategy in the management of PHP during pregnancy. Surgery is recommended in the second trimester when the risk of anaesthesia-induced preterm delivery is lowest and organogenesis is complete [4]. Some authors advocate that all pregnant patients should be offered surgical intervention independent of gestational age [6]. If the diagnosis is made in the 3rd trimester, a discussion with the patient is necessary regarding the risks and benefits of surgery, with some authors advocating that surgery in the 3rd trimester is safe and reduces fetal complications from maternal PHP [30]. Currently with enhanced preoperative localization, improved surgical procedures, and the ability to perform rapid intraoperative serial PTH levels, the operative time can be dramatically reduced [6, 44]. There is only one other prior case report published of the use of intraoperative PTH monitoring to confirm the successful surgical removal of an adenoma in a pregnant patient [45].

### 3.6. Management Based on the Gestational Age of the Fetus

During the first trimester, hypercalcemia should be managed conservatively with medical therapies [6]. Surgical intervention is recommended in the 2nd trimester considering the high cure rate with current safe surgical techniques [6]. Based on the world's largest series of gestational PHP patients, the authors recommend that surgery should be offered early in the second trimester for those with a calcium level >11.4 mg/dL, as pregnancy loss rises exponentially above these levels [15]. Surgery during the 3rd trimester is associated with a higher risk of neonatal complications and mortality [4]; however, is acceptable when the benefit outweighs the risk [30, 46]. If all efforts fail, urgent parathyroidectomy should be recommended regardless of the gestational age of the fetus [47].

## 4. Conclusion

Primary hyperparathyroidism is a rare disorder presenting during pregnancy; however, it represents a preventable cause of fetal and maternal morbidity and mortality. It can be associated with significant maternal and fetal morbidity and mortality, if not recognized and managed appropriately in a timely fashion. In cases of severe symptomatic hypercalcemia related to a parathyroid adenoma, parathyroid surgery in the second trimester is the most definitive treatment and offers the optimal prognosis for both the mother and fetus. Our case highlights the significance of timely recognition and effective management of PHP in pregnancy, leading to optimization of both maternal and fetal outcomes.

## References

[1] Heath H, Hodgson SF, Kennedy MA (1980). Primary hyperparathyroidism. Incidence, morbidity, and potential economic impact in a community. *The New England Journal of Medicine*.

[2] Kohlmeier L, Marcus R (1995). Calcium disorders of pregnancy. *Endocrinology and Metabolism Clinics of North America*.

[3] Armson BA (1994). Parathyroid function and calcium homeostasis. *Infertility & Reproductive Medicine Clinics of North America*.

[4] Carella MJ, Gossain VV (1992). Hyperparathyroidism and pregnancy: case report and review. *Journal of General Internal Medicine*.

[5] Tollin SR (2000). Course and outcome of pregnancy in a patient with mild, asymptomatic, primary hyperparathyroidism diagnosed before conception. *American Journal of the Medical Sciences*.

[6] Schnatz PF, Curry SL (2002). Primary hyperparathyroidism in pregnancy: evidence-based management. *Obstetrical and Gynecological Survey*.

[7] Amaya García MJ, Acosta Feria M, Soto Moreno A (2004). Primary hyperparathyroidism in pregnancy. *Gynecological Endocrinology*.

[8] Pitkin RM, Gebhardt MP (1977). Serum calcium concentrations in human pregnancy. *American Journal of Obstetrics and Gynecology*.

[9] Gelister JSK, Sanderson JD, Chapple CR, O’Riordan JLH, Cowie AGA, Milroy EJG (1989). Management of hyperparathyrodism in pregnancy. *British Journal of Surgery*.

[10] Wagner G, Transbol I, Melchior JC (1964). Hyperparathyroidism and pregnancy. *Acta Endocrinologica*.

[11] Shangold MM, Dor N, Welt SI (1982). Hyperparathyroidism and pregnancy: a review. *Obstetrical and Gynecological Survey*.

[12] Ip P (2003). Neonatal convulsion revealing maternal hyperparathyroidism: an unusual case of late neonatal hypoparathyroidism. *Archives of Gynecology and Obstetrics*.

[13] Kort KC, Schiller HJ, Numann PJ (1999). Hyperparathyroidism and pregnancy. *American Journal of Surgery*.

[14] Delmonico FL, Neer RM, Cosimi AB (1976). Hyperparathyroidism during pregnancy. *American Journal of Surgery*.

[15] Norman J, Politz D, Politz L (2009). Hyperparathyroidism during pregnancy and the effect of rising calcium on pregnancy loss: a call for earlier intervention. *Clinical Endocrinology*.

[16] Kelly TR (1991). Primary hyperparathyroidism during pregnancy. *Surgery*.

[17] Gertner JM, Coustan DR, Kliger AS (1986). Pregnancy as state of physiologic absorptive hypercalciuria. *American Journal of Medicine*.

[18] Lopez JM, Fardella CB (1989). Primary hyperparathyroidism: changes on biochemical and hormonal profile related to pregnancy. *Journal of Endocrinological Investigation*.

[19] Dahlman T, Sjoberg HE, Bucht E (1994). Calcium homeostasis in normal pregnancy and puerperium. A longitudinal study. *Acta Obstetricia et Gynecologica Scandinavica*.

[20] Potts JT (2004). *Diseases of the Parathyroid Gland and Other Hyper- and Hypocalcemic Disorders. Harrison’s Principles of Internal Medicine*.

[21] Hosking DJ (1996). Calcium homeostasis in pregnancy. *Clinical Endocrinology*.

[22] Kent GN, Price RI, Gutteridge DH (1990). Human lactation: forearm trabecular bone loss, increased bone turnover, and renal conservation of calcium and inorganic phosphate with recovery of bone mass following weaning. *Journal of Bone and Mineral Research*.

[23] Kovacs CS, Kronenberg HM (1997). Maternal-fetal calcium and bone metabolism during pregnancy, puerperium, and lactation. *Endocrine Reviews*.

[24] Seely EW, Brown EM, Demaggio DM, Weldon DK, Graves SW (1997). A prospective study of calciotropic hormones in pregnancy and post partum: reciprocal changes in serum intact parathyroid hormone and 1,25-Dihydroxyvitamin D. *American Journal of Obstetrics and Gynecology*.

[25] Zerwekh JE, Breslau NA (1986). Human placental production of 1*α*,25-dihydroxyvitamin D3: biochemical characterization and production in normal subjects and patients with pseudohypoparathyroidism. *Journal of Clinical Endocrinology and Metabolism*.

[26] Seki K, Wada S, Nagata N, Nagata I (1994). Parathyroid hormone-related protein during pregnancy and the perinatal period. *Gynecologic and Obstetric Investigation*.

[27] Kovacs CS, Lanske B, Hunzelman JL, Guo J, Karaplis AC, Kronenberg HM (1996). Parathyroid hormone-related peptide (PTHrP) regulates fetal-placental calcium transport through a receptor distinct from the PTH/PTHrP receptor. *Proceedings of the National Academy of Sciences of the United States of America*.

[28] Kristoffersson A, Dahlgren S, Lithner F, Jarhult J (1985). Primary hyperparathyroidism in pregnancy. *Surgery*.

[29] Reading CC, Charboneau JW, James EM (1982). High-resolution parathyroid sonography. *American Journal of Roentgenology*.

[30] Schnatz PF, Thaxton S (2005). Parathyroidectomy in the third trimester of pregnancy. *Obstetrical and Gynecological Survey*.

[31] Croom RD, Thomas CG (1984). Primary hyperparathyroidism during pregnancy. *Surgery*.

[32] Hong MK, Hsieh CTC, Chen BH, Tu ST, Chou PH (2001). Primary hyperparathyroidism and acute pancreatitis during the third trimester of pregnancy. *Journal of Maternal-Fetal Medicine*.

[33] Clark D, Seeds JW, Cefalo RC (1981). Hyperparathyroid crisis and pregnancy. *American Journal of Obstetrics and Gynecology*.

[34] Negishi H, Kobayashi M, Nishida R (2002). Primary hyperparathyroidism and simultaneous bilateral fracture of the femoral neck during pregnancy. *Journal of Trauma*.

[35] Thomason JL, Sampson MB, Farb HF, Spellacy WN (1981). Pregnancy complicated by concurrent primary hyperparathyroidism and pancreatitis. *Obstetrics and Gynecology*.

[36] Cherry TAD, Kauffman RP, Myles TD (2002). Primary hyperparathyroidism, hypercalcemic crisis and subsequent seizures occurring during pregnancy: a case report. *Journal of Maternal-Fetal and Neonatal Medicine*.

[37] Becker KL, Bilezikian JP, Becker R (2001). *Endocrine Disease in Pregnancy. Principles and Practice of Endocrinology and Metabolism*.

[38] Ludwig GD (1962). Hyperparathyroidism in relation to pregnancy. *The The New England Journal of Medicine*.

[39] Bruce J, Strong JA (1955). Maternal hyperparathyroidism and parathyroid deficiency in the child; with an account of the effect of parathyroidectomy on renal function, and of an attempt to transplant part of the tumour. *The Quarterly Journal of Medicine*.

[40] Cemeroglu AP, Böber E, Büyükgebiz A (2001). Prolonged hypocalcemia in a 2 month-old boy unmasking maternal diagnosis of primary hyperparathyroidism. *Journal of Pediatric Endocrinology and Metabolism*.

[41] Bilezikian JP, Khan AA, Potts JT (2009). Guidelines for the management of asymptomatic primary hyperparathyroidism: summary statement from the third international workshop. *Journal of Clinical Endocrinology and Metabolism*.

[42] Graham EM, Freedman LJ, Forouzan I (1998). Intrauterine growth retardation in a woman with primary hyperparathyroidism: a case report. *Journal of Reproductive Medicine for the Obstetrician and Gynecologist*.

[43] Kleinman GE, Rodriquez H, Good MC, Caudle MR (1991). Hypercalcemic crisis in pregnancy associated with excessive ingestion of calcium carbonate antacid (milk-alkali syndrome): successful treatment with hemodialysis. *Obstetrics and Gynecology*.

[44] Sofferman RA, Standage J, Tang ME (1998). Minimal-access parathyroid surgery using intraoperative parathyroid hormone assay. *Laryngoscope*.

[45] Pothiwala P, Levine SN (2009). Parathyroid surgery in pregnancy: review of the literature and localization by aspiration for parathyroid hormone levels.. *Journal of Perinatology*.

[46] Shnider SM, Webster GM (1965). Maternal and fetal hazards of surgery during pregnancy. *American Journal of Obstetrics and Gynecology*.

[47] Patel NA, Bughi S, Shaw SJ (2005). Hyperparathyroidism in pregnancy. *Endocrinologist*.

